# Adolescent overweight and obesity and the risk of papillary thyroid cancer in adulthood: a large-scale case-control study

**DOI:** 10.1038/s41598-020-59245-3

**Published:** 2020-03-19

**Authors:** Kyoung-Nam Kim, Yunji Hwang, Kyu Hyung Kim, Kyu Eun Lee, Young Joo Park, Su-jin Kim, Hyungju Kwon, Do Joon Park, BeLong Cho, Ho-Chun Choi, Daehee Kang, Sue K. Park

**Affiliations:** 1 Department of Preventive Medicine, Seoul National University College of Medicine, Seoul, Republic of Korea; 2 Division of Public Health and Preventive Medicine, Seoul National University Hospital, Seoul, Republic of Korea; 3 Department of Biomedical Science, Seoul National University Graduate School, Seoul, Republic of Korea; 4 Cancer Research Institute, Seoul National University, Seoul, Republic of Korea; 5 Department of Surgery, Seoul National University Bundang Hospital, Gyeonggi-do, Republic of Korea; 6 Department of Surgery, Seoul National University College of Medicine and Hospital, Seoul, Republic of Korea; 7 Division of Surgery, Thyroid Center, Seoul National University Cancer Hospital, Seoul, Republic of Korea; 8 Department of Internal Medicine, Seoul National University College of Medicine, Seoul, Republic of Korea; 9 Breast and Thyroid Cancer Center, Ewha Womans University College of Medicine, Seoul, Republic of Korea; 10 Department of Family Medicine, Center for Health Promotion and Optimal aging, Seoul National University College of Medicine and Hospital, Seoul, Republic of Korea; 11 Institute of Aging, Seoul National University College of Medicine, Seoul, Republic of Korea; 12 Department of Family Medicine, Healthcare System Gangnam Center, Seoul National University Hospital, Seoul, Republic of Korea

**Keywords:** Cancer epidemiology, Risk factors

## Abstract

This study aimed to investigate the association between adolescent overweight and obesity and PTC risk in adulthood. We conducted a case-control study in the Republic of Korea with 1,549 PTC patients and 15,490 controls individually matched for age and sex. We estimated body mass index (BMI) at age 18 years from self-reported weight at this age. Compared with BMI < 23.0 at age 18 years, BMI ≥ 25.0 at age 18 years was associated with higher PTC risk (odds ratio [OR] = 4.31, 95% confidence interval [CI]: 3.57, 5.22). The association between BMI ≥ 25.0 at age 18 years and PTC risk was stronger among men (OR = 6.65, 95% CI: 4.78, 9.27) than among women (OR = 3.49, 95% CI: 2.74, 4.43), and stronger among individuals with current BMI ≥ 25.0 (OR = 8.21, 95% CI: 6.34, 10.62) than among those with current BMI < 25.0 (OR = 2.21, 95% CI: 1.49, 3.27). Among PTC patients, BMI ≥ 25.0 at age 18 years was associated with extra-thyroidal extension and T stage ≥2, but not with N stage ≥1 or *BRAF*^V600E^ mutation. Adolescent overweight and obesity was associated with higher risk of PTC in adulthood. Our results emphasise the importance of weight management in adolescence to decrease the PTC risk.

## Introduction

Adolescent overweight and obesity is a major public health issue. The prevalence of adolescent overweight and obesity has increased worldwide, including in the United States^1^ and the Republic of Korea^2,3^. Approximately one-fifth of children and adolescents in the United States have obesity^4^. Adolescent overweight and obesity has been associated with higher risk of premature mortality, cardiovascular disease, and cancer in adulthood^5–7^.

Thyroid cancer is most common endocrine cancer in the U.S.^8^ and the Republic of Korea^9^. Although there have been concerns regarding an over-diagnosis and over-treatment of thyroid cancer, previous studies have suggested that the reported increase in thyroid cancer incidence cannot be explained solely by enhanced detection of small tumours owing to the increase in use of ultrasonography and fine-needle aspiration biopsy^10,11^.

Although the reason for the increase in thyroid cancer incidence is still not clear, an increase in the prevalence of adolescent overweight and obesity might be partially responsible. The association between overweight and obesity in adulthood and thyroid cancer risk in later life has been previously reported^12–18^. However, there have been a limited number of studies directly investigating the association between overweight and obesity at age 20 years or younger and thyroid cancer risk afterwards^19–21^, and the results of these studies have been inconsistent.

In the present study, we hypothesised that adolescent overweight and obesity, defined as a body mass index (BMI, kg/m^2^) ≥25.0 at age 18 years, would be associated with higher risk of papillary thyroid cancer (PTC), the most common histologic type of thyroid cancer (>90% of all thyroid cancer cases)^22^. We evaluated this hypothesis using a large-scale case-control study conducted in the Republic of Korea.

## Methods

### Case and control selection

We selected cases from incident thyroid cancer patients admitted at Seoul National University Hospital, Seoul National University Bundang Hospital, and National Medical Centre in the Republic of Korea, between 2010 and 2013. Patients aged ≥20 years who agreed to participate and provided an informed consent were included in the present study. Detailed information on case selection (the Thyroid Cancer Longitudinal Study [T-CALOS] is presented elsewhere^23,24^.

From a total of 1,576 histologically-confirmed PTC patients (the International Classification of Disease for Oncology, 3^rd^ edition: 8050, 8260, 8340–8344, 8350, and 8450–8460), we excluded 27 patients with no information on weight at age 18 years and at enrolment (current) and current height, resulting in 1,549 cases included in the main analyses. We further excluded individuals with no information on extra-thyroidal extension (*n* = 24), thyroid tumour size (*n* = 15), lymph node metastasis (*n* = 116), and *BRAF*^V600E^ mutation (*n* = 248) for analysis of individual associations.

We selected controls from individuals who visited 38 health examination centres for general health examinations, agreed to participate in the study, and provided an informed consent. Detailed information on control selection (the Health Examinees [HEXA] study) is described elsewhere^25^. The study protocol and information obtained during the survey were similar to those for case selection^26^.

From a total of 173,422 eligible participants, we excluded individuals with a history of cancer or thyroid diseases (*n* = 16,578) and those without data on weight at age 18 years and current weight and height (*n* = 32,547); this resulted in 124,297 potential controls. We subsequently performed 1:10 individual matching for age (±5 years) and sex using the greedy matching algorithm of the SAS GMATCH macro^27^.

We conducted the main analyses in 1,549 cases (300 men and 1,249 women) and 15,490 controls (3,000 men and 12,490 women). All study participants in the present study belonged to the same ethnic group (East Asian). The study protocols were designed and followed according to the Declaration of Helsinki^23,25^. The Institutional Review Board of the Seoul National University Hospital approved the study protocols (IRB numbers: 0809-097-258, 1001-067-307, and 1202-088-398). We obtained an informed consent from all study participants.

### Assessment of anthropometric measures

Information on participants’ weight at age 18 years were obtained from an in-person interview by a trained interviewer using a structured questionnaire. Current weight and height were measured at study enrolment by a trained member of the survey staff. We calculated the BMI at age 18 years as self-reported weight (kg) at age 18 years divided by the square of current height (m^2^).

Although the World Health Organization (WHO) suggests BMI cut-offs of <18.5, 18.5–22.9, 23.0–24.9, 25.0–29.9, or ≥30.0 for Asian populations^28^, we combined BMI categories and used the cut-off of <23.0, 23.0–24.9, or ≥25.0, which were not conventional, in further analyses, as the number of participants included in the category of BMI ≥ 30.0 (*n* = 9) was limited and we observed a monotonic association between BMI and PTC risk. We defined adolescent overweight and obesity as a BMI ≥ 25.0 at age 18 years.

In analyses evaluating the associations of height with PTC risk and clinicopathologic features of PTC, we used sex-specific quartiles of height (cm, <165.5, 165.5–169.5, 169.6–173.7, or ≥173.8 for men; <153.1, 153.1–156.9, 157.0–160.2, or ≥160.3 for women) as an independent variable. These cut-offs were arbitrarily chosen considering the characteristics of the research data, because there have been no conventional cut-offs for height.

### Clinicopathologic features of PTC

Information on clinicopathologic features of PTC, such as extra-thyroidal extension, tumour size, lymph node metastasis, and *BRAF*^V600E^ mutation, were obtained from the medical records by a certified medical records officer and confirmed by an endocrine surgeon. The *BRAF*^V600E^ mutation was analysed through DNA amplification by polymerase chain reaction (PCR), purification of DNA amplification product with QIAquick PCR purification kit (Qiagen, Hilden, Germany), and sequencing of DNA with ABI 3130XL Genetic Analyzer BigDye Terminator (Applied Biosystems, Foster City, CA)^23^.

In further analyses, we considered the presence of extra-thyroidal extension (no or yes), T stage (1 or ≥2), N stage (0 or ≥1), and *BRAF*^V600E^ mutation (no or yes).

### Covariates

Based on previous studies on the association between adolescent overweight and obesity and PTC^19–21^, we initially collected the data on age (year), sex, educational level (<high school or ≥high school), tobacco smoking (never-smoker or ever-smoker), alcohol consumption (never-drinker or ever-drinker), history of chronic diseases including type 2 diabetes (no or yes), hypertension (no or yes), and dyslipidaemia (no or yes); data on reproductive factors including menopause (no or yes) were also collected.

Since lifestyle factors did not differ appreciably between cases and controls, we included the afore-mentioned variables, other than tobacco smoking and alcohol consumption, as covariates in further analyses.

### Statistical analysis

Using individually matched case-control pairs, we performed conditional logistic regressions to explore the association between BMI ≥ 25.0 at age 18 years and PTC risk, compared with BMI < 23.0 at age 18 years.

Because previous studies suggested heterogeneity of the association by sex^14,18,21^, we conducted sex-stratified analyses using the same conditional logistic regression models.

Current overweight and obesity may be related to both adolescent overweight and obesity and PTC risk and may confound the association between adolescent overweight and obesity and PTC risk. To confirm that the association between BMI ≥ 25.0 at age 18 years and PTC risk would be independent of current weight status, we conducted stratified analyses by current BMI (≥25.0 vs. <25.0), employing unconditional logistic regression models adjusted for the same covariates. Among various methods to control for the effects of potential confounders, such as stratification, adjustment, and restriction, we used a stratification method, because we wanted to investigate further the heterogeneity of the association between BMI at age 18 year and PTC risk by current BMI.

We assessed the interactions of BMI at age 18 years with sex and current BMI (≥25.0 vs. <25.0) with respect to PTC risk by testing each corresponding product term added to the main logistic regression models.

**Figure 1:**
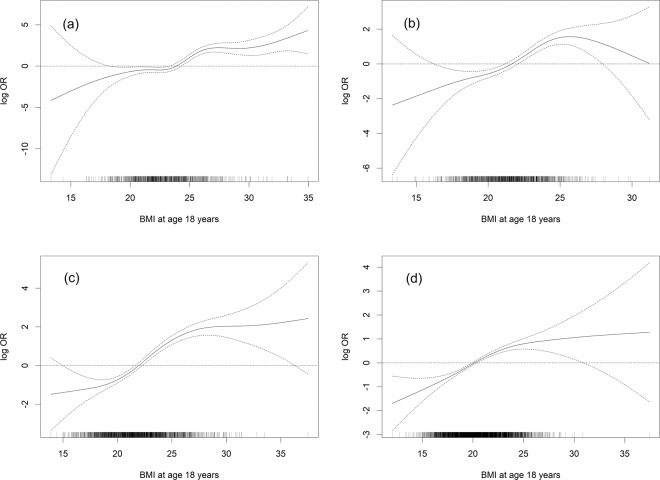
Penalised regression splines for the association between body mass index at age 18 years and the risk of papillary thyroid cancer. (**a**) among men with current body mass index ≥25. (**b**) among men with current body mass index <25. (**c**) among women with current body mass index ≥25. (**d**) among women with current body mass index <25.

By using generalised additive models, we further investigated the associations between BMI (continuous) at age 18 years and PTC risk among men with current BMI ≥ 25, men with current BMI < 25, women with current BMI ≥ 25, and women with current BMI < 25 (Fig. 1).

We conducted unconditional logistic regressions adjusted for the same covariates among the PTC patients to evaluate the associations between BMI at age 18 years and clinicopathologic features of PTC.

Because height is used to calculate BMI and has been associated with PTC risk in previous studies^29,30^, we also repeated all analyses using sex-specific quartiles of height as an independent variable.

We conducted a series of sensitivity analyses after stratifying the study population according to menopausal status in women (premenopausal vs. postmenopausal), birth year (<1950, 1950–1964, or ≥1965), age (<45 years, 45–59 years, or ≥60 years), and history of type 2 diabetes, hypertension, and dyslipidaemia.

We conducted all analyses using SAS version 9.4 (SAS Institute, Inc., Cary, NC) and Stata version 14 (Stata Corp., College Station, TX).

## Results

Age (50.7 years vs. 50.8 years), current BMI (23.7 vs. 23.7), tobacco smoking rate (16.8% vs. 17.3%), and alcohol consumption rate (43.8% vs. 45.0%) were similar among the cases and controls. However, the cases had a higher likelihood of having educational levels ≥high school (85.0% vs. 69.5%) and a history of type 2 diabetes (7.0% vs. 5.2%), hypertension (23.8% vs. 15.8%), and dyslipidaemia (16.3% vs. 7.9%) compared to the controls (Table 1).

**Table 1:** Sociodemographic characteristics of study participants (*n* = 17,039).

	Cases	Controls	*p*-value^a^
	(*n* = 1,549)	(*n* = 15,490)	
Age (year)	50.7 ± 9.4	50.8 ± 9.1	0.71
Height (cm)	161.0 ± 7.5	159.1 ± 7.4	<0.01
Current weight (kg)	61.5 ± 10.2	60.1 ± 9.4	<0.01
Current BMI (kg/m^2^)	23.7 ± 3.1	23.7 ± 2.9	0.54
Sex			
Men	300 (19.4)	3,000 (19.4)	1.00
Women	1,249 (80.6)	12,490 (80.6)	
Educational level			
<High school	232 (15.0)	4,728 (30.5)	<0.01
≥High school	1,317 (85.0)	10,762 (69.5)	
Tobacco smoking			
Never-smoker	1,289 (83.2)	12,812 (83.7)	0.62
Ever-smoker	260 (16.8)	2,678 (17.3)	
Alcohol consumption			
Never-drinker	871 (56.2)	8,521 (55.0)	0.36
Ever-drinker	678 (43.8)	6,969 (45.0)	
History of type 2 diabetes			
No	1,441 (93.0)	14,688 (94.8)	<0.01
Yes	108 (7.0)	802 (5.2)	
History of hypertension			
No	1,181 (76.2)	13,046 (84.2)	<0.01
Yes	368 (23.8)	2,444 (15.8)	
History of dyslipidaemia			
No	1,297 (83.7)	14,261 (92.1)	<0.01
Yes	252 (16.3)	1,229 (7.9)	
Menopause^b^			
No	598 (47.9)	6,337 (50.7)	0.05
Yes	651 (52.1)	6,153 (49.3)	

Abbreviation: BMI, body mass index.

Values are presented as mean ± standard deviation for continuous variables and *n* (%) for categorical variables.

^a^*p*-values were estimated by a t-test for continuous variables and Chi-square test for categorical variables.

^b^Proportions were calculated using the denominator for women.

After adjustment for potential confounders, BMI ≥ 25.0 at age 18 years was found to be associated with higher PTC risk compared with BMI < 23.0 at age 18 years (odds ratio [OR] = 4.31, 95% confidence interval [CI]: 3.57, 5.22). When we categorised BMI according to the WHO criteria for Asians, the results were similar. We also found that greater height was associated with higher PTC risk (*p*-value for trend <0.01) (Table 2).

**Table 2:** Associations^a^ of body mass index at age 18 years and height with the risk of papillary thyroid cancer.

	Cases (*n* = 1,549)	Controls (*n* = 15,490)	OR (95% CI)
	*n* (%)	*n* (%)	
**Body mass index at age 18 years (kg/m**^**2**^**)**			
WHO criteria for Asians			
<18.5	115 (7.4)	2,231 (14.4)	0.54 (0.44, 0.66)
18.5–22.9	956 (61.7)	10,737 (69.3)	Ref.
23.0–24.9	293 (18.9)	1,868 (12.1)	2.02 (1.74, 2.34)
25.0–29.9	176 (11.4)	630 (4.1)	3.98 (3.28, 4.84)
≥30.0	9 (0.6)	24 (0.2)	4.69 (2.07, 10.63)
*p*-for trend			<0.01
**Modified WHO criteria for Asians**			
<23.0	1,071 (69.1)	12,968 (83.7)	Ref.
23.0–24.9	293 (18.9)	1,868 (12.1)	2.19 (1.89, 2.53)
≥25.0	185 (11.9)	654 (4.2)	4.31 (3.57, 5.22)
*p*-for trend			<0.01
Height (cm)^b^			
Quartile 1	269 (17.4)	4,077 (26.3)	Ref.
Quartile 2	247 (16.0)	3,787 (24.5)	0.98 (0.82, 1.18)
Quartile 3	469 (30.3)	3,945 (25.5)	1.79 (1.52, 2.11)
Quartile 4	564 (36.4)	3,681 (23.8)	2.28 (1.93, 2.68)
*p*-for trend			<0.01

Abbreviations: OR, odd ratio; CI, confidential interval; Ref., reference.

^a^Estimated from conditional logistic regression models adjusted for age, sex, educational level, history of type 2 diabetes, hypertension, dyslipidaemia, and menopausal status.

^b^Sex-specific quartiles were used.

When the analyses were stratified by sex, both men and women with BMI ≥ 25.0 at age 18 years have higher PTC risk compared with men and women with BMI < 23.0 at age 18 years (OR = 6.65, 95% CI: 4.76, 9.27 for men; OR = 3.49, 95% CI: 2.74, 4.43 for women). However, the association was found to be stronger among men than women (*p*-value for interaction = 0.01) (Table 3).

**Table 3:** Associations^a^ of body mass index at age 18 years and height with the risk of papillary thyroid cancer, stratified by sex and current body mass index.

	Cases (*n* = 300)	Controls (*n* = 3,000)	OR (95% CI)	Cases (*n* = 1,249)	Controls (*n* = 12,490)	OR (95% CI)	*p*-for interaction
	*n* (%)	*n* (%)		*n* (%)	*n* (%)		
**Stratified by sex**	**Men**			**Women**			
BMI at age 18 (kg/m^2^)							
<23.0	135 (45.0)	2,197 (73.2)	Ref.	936 (74.9)	10,771 (86.2)	Ref.	0.01
23.0–24.9	85 (28.3)	585 (19.5)	2.48 (1.83, 3.35)	208 (16.7)	1,283 (10.3)	2.13 (1.80, 2.52)	
≥25.0	80 (26.7)	218 (7.3)	6.65 (4.78, 9.27)	105 (8.4)	436 (3.5)	3.49 (2.74, 4.43)	
*p*-value for trend			<0.01			<0.01	
Height (cm)							
Quartile 1	46 (15.3)	779 (26.0)	Ref.	223 (17.9)	3,298 (26.4)	Ref.	0.56
Quartile 2	58 (19.3)	763 (25.4)	1.29 (0.85, 1.93)	189 (15.1)	3,024 (24.2)	0.92 (0.75, 1.13)	
Quartile 3	82 (27.3)	749 (25.0)	1.90 (1.29, 2.82)	387 (31.0)	3,196 (25.6)	1.76 (1.47, 2.11)	
Quartile 4	114 (38.0)	709 (23.6)	2.84 (1.94, 4.16)	450 (36.0)	2,972 (23.8)	2.16 (1.80, 2.59)	
*p*-for trend			<0.01			<0.01	
**Current BMI**	≥**25 kg/m**^2^			**<25 kg/m**^2^			
BMI at age 18 (kg/m^2^)							
<23.0	179 (39.9)	3,276 (70.5)	Ref.	892 (81.1)	9,692 (89.4)	Ref.	<0.01
23.0–24.9	117 (26.1)	923 (19.9)	2.77 (2.14, 3.57)	176 (16.0)	945 (8.7)	2.27 (1.89, 2.73)	
≥25.0	153 (34.1)	445 (9.6)	8.21 (6.34, 10.62)	32 (2.9)	209 (1.9)	2.21 (1.49, 3.27)	
*p*-for trend			<0.01			<0.01	
Height (cm)^b^							
Quartile 1	98 (21.8)	1,508 (32.5)	Ref.	171 (15.6)	2,569 (23.7)	Ref.	0.79
Quartile 2	84 (18.7)	1,190 (25.6)	1.04 (0.76, 1.42)	163 (14.8)	2,597 (23.9)	0.92 (0.74, 1.16)	
Quartile 3	133 (29.6)	1,061 (22.9)	1.82 (1.37, 2.42)	336 (30.6)	2,884 (26.6)	1.72 (1.41, 2.10)	
Quartile 4	134 (29.8)	885 (19.1)	2.16 (1.62, 2.90)	430 (39.1)	2,796 (25.8)	2.28 (1.87, 2.77)	
*p*-for trend			<0.01			<0.01	

Abbreviations: OR, odd ratio; CI, confidential interval; BMI, body mass index; Ref., reference.

^a^Estimated from conditional (stratified analyses by sex) and unconditional (stratified analyses by current BMI) logistic regression models for adjusted for age, sex, educational level, history of type 2 diabetes, hypertension, dyslipidaemia, and menopausal status.

^b^Sex-specific quartiles were used.

Stratified analyses by current BMI (≥25.0 vs. <25.0) showed that BMI ≥ 25.0 at age 18 years was associated with higher PTC risk compared with BMI < 23.0 at age 18 years, in both the group with current BMI ≥ 25.0 (OR = 8.21, 95% CI: 6.34, 10.62) and with current BMI < 25.0 (OR = 2.21, 95% CI: 1.49, 3.27). However, the association was stronger in the group with current BMI ≥ 25.0 than in that with current BMI < 25.0 (*p*-value for interaction < 0.01) (Table 3).

In penalised regression spline models, BMI at age 18 years was positively associated with PTC risk among men with current BMI ≥ 25, men with current BMI < 25, women with current BMI ≥ 25, and women with current BMI < 25 (Fig. 1).

We also found that greater height was associated with higher PTC risk among both men and women and among individuals of any current BMI (≥25.0 and <25.0) (all *p*-values for trend <0.01). However, the association did not differ between sex (*p*-value for interaction = 0.56) or current BMI (*p*-value for interaction = 0.79) (Table 3).

Among PTC patients, compared with BMI < 23.0 at age 18 years, BMI ≥ 25.0 at age 18 years was associated with extra-thyroidal extension (OR = 1.50, 95% CI: 1.06, 2.12) and T stage ≥2 (OR = 1.94, 95% CI: 1.03, 3.65), but not with N stage ≥1 (OR = 1.08, 95% CI: 0.64, 1.84) or *BRAF*^V600E^ mutation (OR = 0.82, 95% CI: 0.55, 1.22). Greater height was only associated with N stage ≥1 (*p*-value for trend = 0.01) (Table 4).

**Table 4:** Associations^a^ of body mass index at age 18 years and height with clinicopathologic features (extra-thyroidal extension, T stage, N stage, and *BRAF* mutation) among papillary thyroid cancer patients.

	Clinicopathologic features, *n* (%)		OR (95% CI)	Clinicopathologic features, *n* (%)		OR (95% CI)
	Extra-thyroidal extension			T stage		
	Negative (*n* = 636)	Positive (*n* = 889)		T1 (*n* = 1,454)	T2+ (*n* = 80)	
BMI at age 18 (kg/m^2^)						
<23.0	456 (71.7)	596 (67.0)	Ref.	1,015 (69.8)	45 (56.3)	Ref.
23.0–24.9	118 (18.6)	170 (19.1)	1.06 (0.81, 1.39)	271 (18.6)	19 (23.8)	1.49 (0.84, 2.63)
≥25.0	62 (9.8)	123 (13.8)	1.50 (1.06, 2.12)	168 (11.6)	16 (20.0)	1.94 (1.03, 3.65)
*p*-for trend			0.04			0.03
Height (cm)^b^						
Quartile 1	97 (15.3)	170 (19.1)	Ref.	252 (17.3)	16 (20.0)	Ref.
Quartile 2	99 (15.6)	143 (16.1)	0.87 (0.61, 1.26)	229 (15.8)	16 (20.0)	1.12 (0.54, 2.32)
Quartile 3	203 (31.9)	258 (29.0)	0.79 (0.58, 1.09)	444 (30.5)	18 (22.5)	0.69 (0.34, 1.41)
Quartile 4	237 (37.3)	318 (35.8)	0.90 (0.65, 1.24)	529 (36.4)	30 (37.5)	1.02 (0.52, 1.99)
*p*-for trend			0.55			0.82
	***BRAF*** **mutation**			**N stage**		
	*BRAF*^wt^ (*n* = 400)	*BRAF*^V600E^ (*n* = 90)		N0 (*n* = 1,160)	N1 + (*n* = 163)	
BMI at age 18 (kg/m^2^)						
<23.0	285 (71.3)	629 (69.8)	Ref.	817 (70.4)	115 (70.6)	Ref.
23.0–24.9	69 (17.3)	170 (18.9)	0.99 (0.72, 1.37)	217 (18.7)	25 (15.3)	0.78 (0.49, 1.25)
≥25.0	46 (11.5)	102 (11.3)	0.82 (0.55, 1.22)	126 (10.9)	23 (14.1)	1.08 (0.64, 1.84)
*p*-for trend			0.39			0.90
Height (cm)^b^						
Quartile 1	200 (17.2)	25 (15.3)	Ref.	82 (20.5)	141 (15.7)	Ref.
Quartile 2	186 (16.0)	23 (14.1)	0.87 (0.47, 1.61)	68 (17.0)	134 (14.9)	1.16 (0.77, 1.74)
Quartile 3	363 (31.3)	43 (26.4)	0.87 (0.51, 1.49)	113 (28.3)	280 (31.1)	1.50 (1.05, 2.15)
Quartile 4	411 (35.4)	72 (44.2)	1.25 (0.74, 2.10)	137 (34.3)	346 (38.4)	1.54 (1.07, 2.20)
*p*-for trend			0.25			0.01

Abbreviations: OR, odd ratio; CI, confidential interval; Ref., reference.

^a^Estimated from unconditional logistic regression models adjusted for age, sex, educational level, history of type 2 diabetes, hypertension, dyslipidaemia, and menopausal status.

^b^Sex-specific quartiles were used.

We conducted several sensitivity analyses. The results did not change appreciably in each stratum on evaluating the association between BMI at age 18 years and PTC risk stratified by menopausal status in women (premenopausal vs. postmenopausal) (data not shown), birth year (<1950, 1950–1964, or ≥1965), age (<45 years, 45–59 years, and ≥60 years) (Supplementary Table 1), history of type 2 diabetes, hypertension, or dyslipidaemia (Supplementary Table 2).

## Discussion

In the present study, we found an association between adolescent overweight and obesity and higher PTC risk in adulthood. The association was found to be stronger among men and among individuals with current BMI ≥ 25, compared with women and those with current BMI < 25. Among PTC patients, adolescent overweight and obesity was associated with extra-thyroidal extension and larger tumour size of PTC.

There have been a limited number of studies investigating the association between body weight in early life and thyroid cancer risk in adulthood, and the results of these studies are inconsistent. In a case-control study conducted in Japan, BMI at age 20 years was associated with higher risk of thyroid cancer in later life^19^. In a cohort study in Denmark, BMI at age 7–13 years was associated with higher thyroid cancer risk in adulthood^21^. However, in a case-control study in Serbia, BMI in childhood or adolescence was not associated with the risk of PTC among cases and controls younger than age 20 years^20^. The results of these studies suggest that overweight and obesity in early life might be associated with PTC risk in adulthood but not with risk of early-onset PTC, defined as PTC development in those younger than 20 years of age.

Previous studies examining the association between body weight in early life and thyroid cancer risk in adulthood^19,21^, as well as those exploring the association between overweight and obesity in adulthood and thyroid cancer risk in later life^14,18^, have reported a stronger association among men than among women. These results, which are consistent with the results of the present study, suggest that imbalance of sex hormones may contribute to the observed association between overweight and obesity and PTC risk^31,32^.

We found that adolescent overweight and obesity was associated with higher PTC risk not only among individuals with current BMI ≥ 25.0, but also among those with current BMI < 25.0. These results suggest that adolescent overweight and obesity may increase the PTC risk even among individuals who are no longer overweight or have obesity when they reach adulthood and emphasise the importance of maintaining proper body weight in adolescence with respect to the PTC risk. However, the point estimate for the association between BMI at age 18 years and PTC risk was substantially lower among those with current BMI < 25.0 than those with current BMI ≥ 25.0, implying that PTC risk might be lowered by controlling body weight during adulthood even among individuals who were overweight or had obesity in adolescence.

Adolescent overweight and obesity was found to be associated with extra-thyroidal extension and larger tumour size, suggesting aggressiveness and poor prognosis. Previous studies have reported that BMI in adulthood is associated with extra-thyroidal extension, advanced stage of PTC^33^, and postoperative locoregional events^34^ among PTC patients. However, to the best of our knowledge, no previous study has investigated the association between adolescent overweight and clinicopathologic features of PTC. Further studies, including those employing a cohort design, are warranted to confirm these results.

Previous studies^13,21,29^, although not all^12^, have reported that greater height is associated with higher risk of thyroid cancer, which is consistent with the results of the present study. The positive association between greater height and PTC risk may be due to, at least in part, shared biological processes of growth hormone and insulin-like growth factor, which could promote both postnatal growth and mutation, proliferation, adhesion, and migration of thyroid cells^30,35,36^. However, the associations between greater height and clinicopathologic features of PTC have not been investigated in previous studies. Further studies are needed to confirm the results of the present study, such as the association between greater height and N stage ≥1.

Several biological pathways can be responsible for the association between adolescent overweight and obesity and PTC risk (Supplementary Fig. 1). Adolescent overweight and obesity can accelerate growth by increasing growth hormone or insulin-like growth factor 1, and can induce imbalance of hormones such as insulin, leptin, and thyroid stimulating hormone^37^; this can lead to higher risk of thyroid carcinogenesis^35,38^. Adolescent overweight and obesity may also induce an imbalance in the estrogen level, which can activate mitogen-activated protein kinase and other growth factors, affecting the development and aggressiveness of thyroid cancer^31,32^. In addition, chronic subclinical inflammation of the adipose tissue and subsequent local inflammation of the thyroid may also contribute to thyroid cancer development^39^.

The present study has some limitations. First, there is a possibility that adolescents who are overweight or have obesity are more likely to receive medical care (including thyroid screening) and be included in the case group due to differences in detection rate. Second, we obtained the data on weight at age 18 years from self-reports; information bias could have occurred. However, a systematic difference in weight at age 18 years recalled by the PTC patients and that recalled by the control group is unlikely. Third, the results of the present study should be cautiously generalised to other ethnic populations due to the differences in body size and reported heterogeneous associations between weight and other health outcomes, such as mortality, between ethnic groups^40^.

However, the present study also has some strengths. First, we collected comprehensive data through standardised operating procedures. We collected the epidemiological data through a face-to-face interview using a structured questionnaire and the clinical data through a medical chart review. Second, we obtained data on a sufficient number of male PTC patients (*n* = 300) to analyse the association among male subgroups; this has not been extensively investigated to date. Although this is a case-control study and the male to female ratio in the present study did not reflect the male to female ratio of papillary thyroid cancer in the population precisely, papillary thyroid cancer is reported to be more common among women than men not only worldwide including Asia, north America, and Europe^41^. Third, we conducted several analyses on the association between adolescent overweight and the clinicopathologic features of PTC related to tumour aggressiveness, which also had not been previously investigated.

## Conclusion

We found that adolescent overweight and obesity was associated with higher risk of PTC. The association was stronger among men than women and stronger among individuals with current BMI ≥ 25 than those with current BMI < 25. Adolescent overweight and obesity was also associated with higher tumour aggressiveness among PTC patients. The results of the present study provide additional evidence that public health concerns and policy intervention are needed for weight management in adolescence to decrease the PTC risk.

## Supplementary information

Supplementary information

## Data Availability

The datasets generated and/or analysed during the current study are available from the corresponding author on reasonable request.
