# Supplementary material for: Adolescent overweight and obesity and the risk of papillary thyroid cancer in adulthood: a large-scale case-control study
Source: Sci Rep. 2020 Mar 19;10:5000. doi: 10.1038/s41598-020-59245-3 (PMC7081310; doi:10.1038/s41598-020-59245-3)

**Supplementary Material**

**Adolescent overweight and obesity and the risk of papillary thyroid cancer in adulthood: a large-scale case-control study**

Kyoung-Nam Kim, Yunji Hwang, Kyu Hyung Kim, Kyu Eun Lee, Young Joo Park, Su-jin Kim, Hyungju Kwon, Do Joon Park, BeLong Cho, Ho-Chun Choi, Daehee Kang, and Sue K. Park

**Table of Contents**

**Supplementary Table 1.** Association between body mass index at age 18 years and the risk of papillary thyroid cancer by birth year

**Supplementary Table 2.** Association between body mass index at age 18 years and the risk of papillary thyroid cancer, stratified by chronic diseases (type 2 diabetes, hypertension, and dyslipidemia)
**Supplementary Fig. 1.** Biological pathways for the association between adolescent overweight and obesity and papillary thyroid cancer risk

**Supplementary Table 1.** Association^a^ between body mass index at age 18 years and the risk of papillary thyroid cancer by birth year and age

|  | **Body mass index at age 18 years (kg/m^2^)** | | |  |
| --- | --- | --- | --- | --- |
|  | < 23.0 | 23.0–24.9 | ≥25.0 |  |
|  | OR (95% CI) | OR (95% CI) | OR (95% CI) | *p*-for trend |
| **Birth year** |  |  |  |  |
| <1950 | Ref. | 2.62 (1.84, 3.73) | 3.48 (2.30, 5.25) | <0.01 |
| 1950–1964 | Ref. | 2.39 (1.94, 2.94) | 4.54 (3.38, 6.11) | <0.01 |
| ≥1965 | Ref. | 1.74 (1.33, 2.27) | 5.28 (3.77, 7.40) | <0.01 |
| **Age (years)** |  |  |  |  |
| <45 | Ref. | 1.73 (1.28, 2.34) | 5.62 (3.96, 7.97) | <0.01 |
| 45–59 | Ref. | 2.39 (1.96, 2.92) | 4.42 (3.31, 5.90) | <0.01 |
| ≥60 | Ref. | 2.31 (1.70, 3.14) | 3.32 (2.32, 4.77) | <0.01 |

Abbreviations: OR, odd ratio; CI, confidential interval; Ref., reference

^a^Estimated from unconditional logistic regression models adjusted for age, sex, educational level, history of diabetes, hypertension, dyslipidaemia, and menopausal status.

**Supplementary Table 2.** Association^a^ between body mass index at age 18 years and the risk of papillary thyroid cancer, stratified by chronic diseases (type 2 diabetes, hypertension, and dyslipidemia) status

| BMI at age 18 (kg/m^2^) | Cases | Controls |  | Cases | Controls |  |  |
| --- | --- | --- | --- | --- | --- | --- | --- |
|  | *n* (%) | *n* (%) | OR (95% CI) | *n* (%) | *n* (%) | OR (95% CI) | *p*-for interaction |
|  | **No type 2 diabetes** | | | **Type 2 diabetes** | | |  |
| <23.0 | 1,020 (70.8) | 12,369 (84.2) | Ref. |  |  | Ref. | 0.30 |
| 23.0–24.9 | 264 (18.3) | 1,727 (11.8) | 2.18 (1.88, 2.54) | 51 (47.2) | 596 (74.3) | 2.56 (1.53, 4.29) |  |
| ≥25.0 | 157 (10.9) | 589 (4.0) | 4.21 (3.45, 5.14) | 29 (26.9) | 141 (17.6) | 6.55 (3.66, 11.70) |  |
| *p*-for trend |  |  | <0.01 | 28 (25.9) | 65 (8.1) | <0.01 |  |
|  |  |  |  |  |  |  |  |
|  | **No hypertension** | | | **Hypertension** | | |  |
| <23.0 | 868 (73.5) | 11,090 (85.0) | Ref. | 203 (55.2) | 1,878 (76.8) | Ref. | 0.22 |
| 23.0–24.9 | 201 (17.0) | 1,483 (11.4) | 2.12 (1.79, 2.52) | 92 (25.0) | 385 (15.8) | 2.45 (1.85, 3.26) |  |
| ≥25.0 | 112 (9.5) | 473 (3.6) | 4.17 (3.31, 5.25) | 73 (19.8) | 181 (7.4) | 4.82 (3.46, 6.71) |  |
| *p*-for trend |  |  | <0.01 |  |  | <0.01 |  |
|  |  |  |  |  |  |  |  |
|  | **No dyslipidemia** | | | **Dyslipidemia** | | |  |
| <23.0 | 908 (70.0) | 11,953 (83.8) | Ref. | 163 (64.7) | 1,015 (82.6) | Ref. | 0.62 |
| 23.0-24.9 | 242 (18.7) | 1,712 (12.0) | 2.19 (1.87, 2.56) | 51 (20.2) | 156 (12.7) | 2.22 (1.53, 3.23) |  |
| ≥25.0 | 147 (11.3) | 596 (4.2) | 4.26 (3.46, 5.23) | 38 (15.1) | 58 (4.7) | 5.14 (3.20, 8.25) |  |
| *p*-for trend |  |  | <0.01 |  |  | <0.01 |  |

Abbreviations: BMI, body mass index; OR, odd ratio; CI, confidential interval; Ref., reference

^a^Estimated from unconditional logistic regression models adjusted for age, sex, educational level, history of diabetes, hypertension, dyslipidaemia, and menopausal status.

**Figure Legend**

**Supplementary Fig. 1.** Biological pathways for the association between adolescent overweight and obesity and papillary thyroid cancer risk


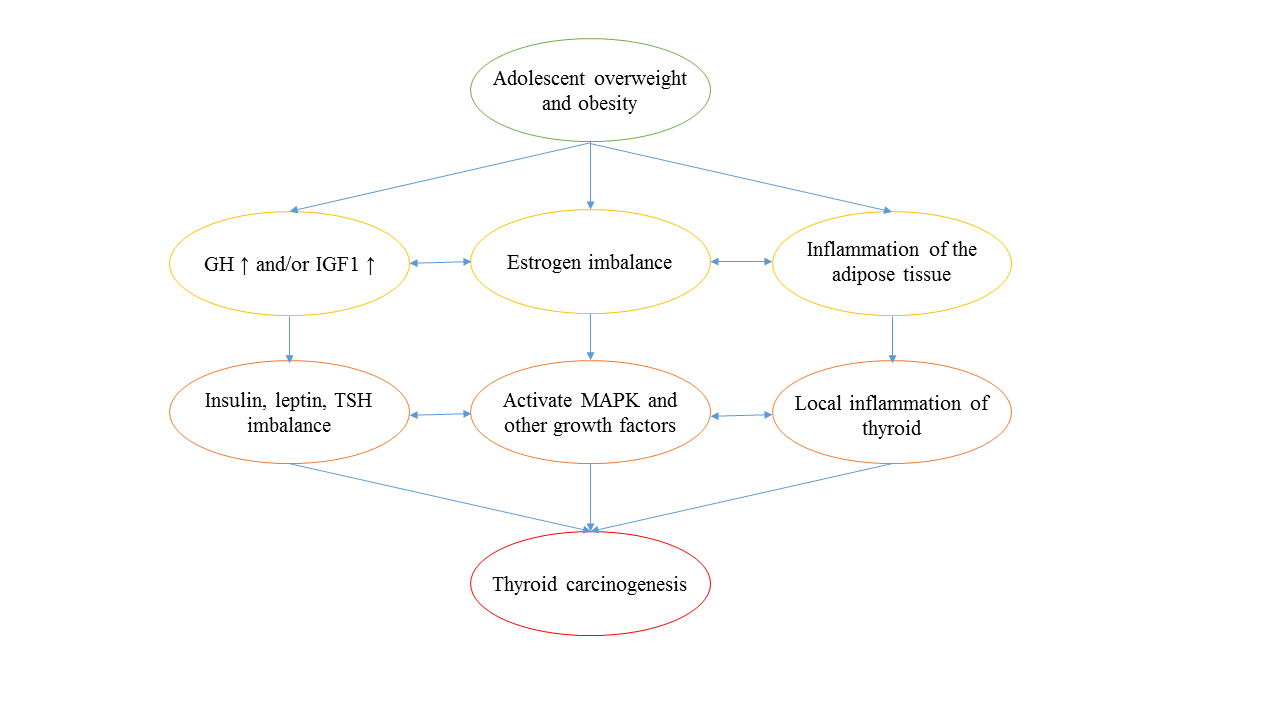

Supplement: Supplementary file 1 — Supplementary information [file 41598_2020_59245_MOESM1_ESM.docx]
